# Striking reduction of amyloid plaque burden in an Alzheimer's mouse model after chronic administration of carmustine

**DOI:** 10.1186/1741-7015-11-81

**Published:** 2013-03-26

**Authors:** Crystal D Hayes, Debleena Dey, Juan Pablo Palavicini, Hongjie Wang, Kshitij A Patkar, Dimitriy Minond, Adel Nefzi, Madepalli K Lakshmana

**Affiliations:** 1 Section of Neurobiology, Torrey Pines Institute for Molecular Studies, SW Village Parkway 11350, Port Saint Lucie, FL 34987, USA; 2 Department of Chemistry, Torrey Pines Institute for Molecular Studies, SW Village Parkway 11350, Port Saint Lucie, FL 34987, USA; 3 Peptide-based Therapeutics, Torrey Pines Institute for Molecular Studies, SW Village Parkway 11350, Port Saint Lucie, FL 34987, USA

**Keywords:** amyloid-β, amyloid plaques, carmustine, 1, 3 bis (2-chloroethyl)-1-nitrosourea, cytotoxicity, MTT assay, LDH release

## Abstract

**Background:**

Currently available therapies for Alzheimer's disease (AD) do not treat the underlying cause of AD. Anecdotal observations in nursing homes from multiple studies strongly suggest an inverse relationship between cancer and AD. Therefore, we reasoned that oncology drugs may be effective against AD.

**Methods:**

We screened a library of all the FDA-approved oncology drugs and identified bis-chloroethylnitrosourea (BCNU or carmustine) as an effective amyloid beta (Aβ) reducing compound. To quantify Aβ levels, Chinese hamster ovary (CHO) cells stably expressing amyloid precursor protein 751WT (APP751WT) called 7WD10 cells were exposed to different concentrations of BCNU for 48 hours and the conditioned media were collected. To detect Aβ the conditioned media were immunoprecipitated with Ab9 antibody and subjected to immunoblot detection. Amyloid plaques were quantified in the brains of a mouse model of AD after chronic exposure to BCNU by thoflavin S staining.

**Results:**

BCNU decreased normalized levels of Aβ starting from 5 μM by 39% (*P *< 0.05), 10 μM by 51% (*P *< 0.01) and 20 μM by 63% (*P *< 0.01) in CHO cells compared to a control group treated with butyl amine, a structural derivative of BCNU. Interestingly, soluble amyloid precursor protein α (sAPPα) levels were increased to 167% (*P *< 0.01) at 0.5 μM, 186% (*P *< 0.05) at 1 μM, 204% (*P *< 0.01) at 5 μM and 152% (*P *< 0.05) at 10 μM compared to untreated cells. We also tested the effects of 12 structural derivatives of BCNU on Aβ levels, but none of them were as potent as BCNU. BCNU treatment at 5 μM led to an accumulation of immature APP at the cell surface resulting in an increased ratio of surface to total APP by 184% for immature APP, but no change in mature APP. It is also remarkable that BCNU reduced Aβ generation independent of secretases which were not altered up to 40 μM. Interestingly, levels of transforming growth factor beta (TGFβ) were increased at 5 μM (43%, *P *< 0.05), 10 μM (73%, *P *< 0.01) and 20 μM (92%, *P *< 0.001). Most significantly, cell culture results were confirmed *in vivo *after chronic administration of BCNU at 0.5 mg/kg which led to the reduction of Aβ40 by 75% and amyloid plaque burden by 81%. Conversely, the levels of sAPPα were increased by 45%.

**Conclusions:**

BCNU reduces Aβ generation and plaque burden at non-toxic concentrations possibly through altered intracellular trafficking and processing of APP. Taken together these data provided unequivocal evidence that BCNU is a potent secretase-sparing anti-Aβ drug.

See related commentary article here http://www.biomedcentral.com/1741-7015/11/82

## Background

Alzheimer's disease (AD) is a devastating neurodegenerative disorder of older persons presented with progressive intellectual deterioration involving memory, language, judgment and problem solving ultimately leading to a total dependence on nursing care. Recent statistics suggest that nearly 35.6 million patients are affected by AD worldwide and that about 4.6 million new cases are added each year causing an enormous social and economic burden [1]. While death rates due to stroke, heart disease and cancer have a decreasing trend, deaths related to AD have actually increased by 66% between 2000 and 2008 [2]. AD is responsible for causing more than 100,000 deaths each year with a total annual cost of care and treatment exceeding $100 billion in the United States alone [2,3]. Accumulation of amyloid plaques composed of amyloid β peptide (Aβ), derived from amyloid precursor protein (APP) by consecutive actions of β- and γ-secretases is a major hallmark of AD. Although the causal relationship between Aβ and AD is not firmly established, increasing genetic, biochemical and pathological evidence strongly implies that Aβ has an early and crucial role in AD pathogenesis [4]. Therefore, most research efforts are now focused on reducing the levels of Aβ. More specifically, the biogenesis of Aβ has been the prime validated drug target for AD. With the failure of anti-amyloid therapy to improve cognitive measures in clinical trials including the most recent (semagacestat [5,6], homotaurine [7] tarenfurbil [8] and bapineuzumab [9]), the so called amyloid 'naysayers' are vocally suggesting that anti-amyloid therapeutic approaches be abandoned. However, improved cognitive measures that correlate well with decreased Aβ levels, even up to 4.5 years after the initial vaccination with Aβ in clinical trials [10,11], strongly support Aβ's causal role in AD and clearly suggest that reducing Aβ levels in the brains of AD patients is an effective approach for successful therapy. Moreover, accelerated cortical atrophy continues to be well correlated with high Aβ deposition in multiple studies which used the most advanced technology and tracing compounds, further strengthening the pivotal role of Aβ in AD [12,13].

At present, the number of therapeutic options for AD remains severely limited. Currently, there are five US Food and Drug Administration (FDA)-approved drugs available for the treatment of AD which target either increasing cholinergic transmission or reducing glutamatergic transmission. None of them can stop or even slow down the underlying neurodegenerative process because there is an extensive multifocal neurodegeneration occurring due to accumulation of Aβ. These drugs do not alter this underlying cause. So far, nearly 1,000 clinical trials have been attempted throughout the world to validate an effective therapy for AD based on all possible mechanisms of action including, in the most recent years, both β- and γ-secretase inhibitors [14]. The classes of drugs being investigated include growth factors, Ca2+ antagonists, intravenous immunoglobulin, alpha secretase stimulators, Aβ oligomer inhibitors, metal chelators, neuroprotective agents, cholesterol lowering drugs, anti-inflammatory agents, anti-epileptics and immunization [15]. Unfortunately, instead of improving their cognitive measures, many patients experienced worsening of the symptoms and some patients even developed skin cancer after treatment with semagacestat, a γ-secretase inhibitor [5,6]. The most likely explanation for the failure of γ-secretase inhibitors in the clinical trials is because γ-secretase has dozens of target proteins particularly notch signaling which is crucial for cell-cell communication, immune system formation and cell proliferation and survival [16]. For β-secretase (BACE), in addition to having dozens of other substrates, the active site is so large that only compounds with more than 500 molecular weight may efficiently inhibit the enzyme, but then they are unable to pass through the blood-brain barrier (BBB) [17]. More importantly, multiple labs have reported that BACE1 deficiency in genetically engineered mice is associated with impaired learning [18], further defeating the very purpose for which it is intended. Although effective BACE inhibitors have been shown in recent years to lower Aβ levels in animal models [19,20], overall, secretase inhibitors have so far failed to demonstrate the expected efficacy against AD [6]. Therefore, alternative targets that may modulate Aβ generation, preferably without directly inhibiting γ-secretase or BACE, are very important at this juncture.

Significantly, Aβ has been implicated to play a vital role in the pathogenesis of not only AD but also traumatic brain injury (TBI) [21], cerebral amyloid angiopathy (CAA) [22], glaucoma [23] and Niemann-Pick type C disease [24]. As with AD, there are no effective prevention or treatment strategies for these disorders. Therefore, development of any safe secretase-sparing anti-Aβ drug would have wider clinical applications.

It is also important to note that there is a strong relationship between cancer and AD. For instance, a recent population based cohort study revealed a 43% lower risk of ever developing AD among cancer patients [25]. Similarly, patients diagnosed with AD showed a 69% lower risk of developing cancer [25]. These observations have been confirmed by longitudinal prospective studies [26]. Because of the solid relationship between AD and cancer, we speculated that anticancer drugs may exert favorable effects on AD. Therefore, we decided to screen all FDA-approved oncology drugs in a cell-based assay and identified bis-chloroethylnitrosourea (BCNU or carmustine) as a potential anti-Aβ drug.

Here we show for the first time that BCNU-exposed Chinese hamster ovary (CHO) cells have significantly reduced levels of amyloid β peptide (Aβ) and c-terminal fragments (CTFs) in cell cultures. Also, chronic administration of BCNU for 60 days in a mouse model of AD robustly decreased levels of Aβ40 and amyloid plaque burden at six months of age. More importantly, Aβ levels and plaque burden were reduced at non-toxic concentrations of BCNU independent of secretases suggesting that BCNU may be an effective Aβ lowering, disease-modifying drug against AD.

## Methods

### Chemicals and antibodies

The FDA-approved oncology drug set II was obtained from the open chemical repository of the National Cancer Institute/National Institutes of Health Developmental Therapeutics Program (NCI/NIH DTP) [27]. 1, 3 bis (2-chloroethyl)-1-nitrosourea (BCNU) or carmustine (cat# C0400), thioflavin S (cat #T1892), ethylene glycol tetraacetic acid (EGTA) (cat # E4378), paraformaldehyde (PFA) (cat # P6148), dimethyl sulfoxide (DMSO) (cat # 472301) and glutaraldehyde (cat # G-7776) were all purchased from Sigma-Aldrich (St. Louis, MO, USA). EZ-Link Sulfo-NHS-LC-biotin was from Pierce (Rockford, IL, USA). The monoclonal antibody Ab9 used for immunoprecipitation of Aβ was purified from supernatants of the hybridoma generated in mice by Biomatik Corporation (Ontario, ON, Canada). The polyclonal antibody CT15 (against C-terminal 15 residues of APP) and the polyclonal antibody, 63G (against mid region of APP) have been described previously [28,29]. The monoclonal antibody 6E10 (cat # SIG-39300, recognizing 1- 17 of Aβ sequence) was obtained from Covance Research (Denver, CO, USA). Polyclonal anti-sAPPβ-WT antibody (cat # 18957) was purchased from IBL Co. Ltd (Gunma, Japan). Monoclonal anti-Iba1-AIF1 antibody (cat # MABN92) was purchased from Millipore (Billerica, MA, USA). Polyclonal anti-TGF beta 1 antibody (cat # NBP1-67698) was purchased from Novus Biologicals (Littleton, CO, USA). Mouse monoclonal antibody against beta-actin (cat # A00702) was purchased from Genscript USA Inc. (Piscataway, NJ, USA). Secondary antibodies, such as peroxidase-conjugated AffiniPure goat anti-mouse (Code # 115-035-146) and ant-rabbit (code # 111-035-144) immunoglobulin Gs (IgGs), were purchased from Jackson ImmunoResearch Laboratories (West Grove, PA, USA). Anti-mouse IgG and anti-rabbit IgG-agarose beads were from American Qualex International (San Clemente, CA, USA). ADAM10 and 17 enzymes, and the substrate for inhibition assays were purchased from R&D Systems (Minneapolis, MN, USA; cat#: 936-AD-020, 930-ADB-010, ES010, respectively).

### Quantitation of Aβ, CTFs and sAPPs in 7WD10 cells

The methods for the generation and characterization of CHO cells stably expressing APP751wt (7WD10 cells) for the secretion of Aβ into the conditioned medium (CM) have been described previously [28,29]. To immunoprecipitate Aβ, 7WD10 cells were grown in six-well plates and were treated with BCNU at final concentrations of 0, 0.5, 1.0, 5.0, 10.0 and 20.0 μM in duplicate wells. After 48 hours, the CM was collected, centrifuged to remove cell debris and immunoprecipitated overnight using a monoclonal Ab9 antibody (recognizes 1 - 16 amino acids of Aβ) to pull-down total Aβ. After SDS-PAGE electrophoresis using NuPAGE 4% to 12% bis-tris gels, total Aβ was detected by immunoblotting using a mixture of 6E10/82E1 antibodies which reliably detects total Aβ as described previously [28,29]. The CM was also immunoblotted to detect sAPPα (6E10), sAPPβ (anti-sAPPβ-wt rabbit IgG from IBL America Ltd) and sAPPtotal (63G) using the indicated antibodies. To detect APP holoprotein and CTFs (CT15 antibody raised against the last 15 amino acids of APP), the cells were lysed using lysis buffer (1% Nonidet P-40) with complete protease inhibitor mix (Sigma) and equal amounts of proteins were loaded into each well and subjected to SDS-PAGE electrophoresis. Following transfer onto polyvinylidene difluoride (PVDF) membranes, they were blocked with 5% milk in Tris-buffered saline with Tween (TBS-T) and incubated overnight with primary antibodies followed by one to four hours of incubation with horseradish peroxidase (HRP)-conjugated secondary antibodies, such as monoclonal mouse anti-goat IgG light chain or monoclonal mouse anti-rabbit IgG light chain. The protein signals were detected using Super Signal West Pico Chemiluminescent substrate (Pierce). Quantitation of Western blot signals was done using Java-based ImageJ software available freely from NIH.

### APP turn-over and surface biotinylation experiments

Near confluent 7WD10 cells in duplicate wells were treated with BCNU at 10.0 mM concentration and after 24 hours, washed two times with cold PBS and incubated with 2.0 mg/ml sulfo-NHS-LC-biotin in PBS, pH 8.0 under ultra-low shaking on ice in the cold room. After an hour of incubation, cells were washed three times in PBS and lysates were prepared using 1% Nonidet P-40 lysis buffer containing complete protease mix as described above. Biotinylated proteins were pulled-down by immunoprecipitation with anti-biotin antibody plus anti-mouse agarose beads. The samples were subjected to SDS-PAGE and APP was detected with CT15 antibody. To assess the effect of BCNU on APP stability, cycloheximide experiments were done essentially as previously described in our published papers [28,29]. Briefly, 7WD10 cells were incubated with cycloheximide at a concentration of 100 mg/ml in PBS at 37^°^C in a CO_2 _incubator. Treated and untreated cells were harvested and lysed at 0, 15, 30, 60, and 120 minutes. The lysates were processed to detect APP with CT15 antibody as described above.

### Measurement of activities of secretase enzymes

Enzyme activities were measured using partially purified enzymes from mouse brain homogenates using commercially available kits for BACE (cat # 565785, EMD Millipore, a division of Merck KGaA, Darmstadt, Germany) and γ-secretase (cat # FP003, R&D Systems) according to the manufacturer's instructions. The fluorogenic substrate used for BACE was glu-val-lys-met-asp-ala-glu-phe-lys, and for γ-secretase was NMA-GGVVIATVK (DNP)-DRDRDR-NH2. The procedure briefly includes membrane isolations from the mouse brains at 4^°^C by homogenization using an extraction buffer (20 mM HEPES, pH 7.5; 50 mM KCl and 2 mM EGTA). Lysates were centrifuged at 800 g for 10 minutes to remove nuclei and large cell debris in the pellet and the supernatants collected. The pellets were re-homogenized and more supernatants were collected in the same way. The resulting supernatants were pooled and centrifuged at 100,000 g for 1 hour at 4^°^C. The resulting membrane pellet was washed once in extraction buffer and suspended in the same buffer plus 10% glycerol and flash-frozen in liquid nitrogen and stored at -80^°^C until used. The total protein concentrations in membrane preparations were determined using the BCA method (Pierce). Membranes were resuspended at 0.5 mg/ml concentrations in resuspension buffer and solubilized at 4°C for one hour with end-over-end rotation. Following centrifugation at 100,000 g for one hour, the supernatants were collected.

The assay mixture consisted of 50 ul of partially purified enzyme preparation, 48 ul of 2 times reaction buffer (20 mM HEPES, pH, 7.0; 150 mM of KCl; 2 mM EGTA; 1% (W/V) CHAPSO) and 2 ul of BACE or γ-secretase substrate. The mixture was incubated for two hours in the dark and fluorescence was read at 320/420 nm for BACE and 355/440 nm for γ-secretase. Some samples also included BACE or γ-secretase specific inhibitors to confirm the specificity of enzyme activity. One negative control without lysate and another without substrate were included. The positive control included was 2.0 ul of recombinant BACE enzyme. The enzyme activity was calculated per mg protein and was expressed as percentage change in BCNU-treated samples from untreated controls.

ADAM10 and 17 inhibition assays followed the same general protocol: 5 μL of 3x enzyme solution (3 and 30 nM) in assay buffer (10 mM HEPES, 0.001% Brij-35, pH 7.5) were added to solid bottom white 384 low volume plates (Nunc cat# 264706). Next, 5 μL of test compounds or pharmacological controls were added to corresponding wells. After 30 minutes incubation at room temperature (RT) the reactions were started by addition of 5 μL of 3x solutions of substrate (30 μM). Fluorescence was measured every 30 minutes for 2 hours using the multimode microplate reader Synergy H4 (Biotek Instruments, Winooski, VT, USA) using λ_excitation _= 324 nm and λ_emission _= 405 nm. Rates of hydrolysis were obtained from plots of fluorescence versus time, and inhibition was calculated using rates obtained from wells containing substrate only (100% inhibition) and substrate with enzyme (0% inhibition). The IC_50 _value of the pharmacological control ((N-hydroxy-1-(4-methoxyphenyl)sulfonyl-4-(4-biphenylcarbonyl)piperazine-2-carboxamide, Calbiochem cat#: 444252) was also calculated to ascertain the assay robustness.

### Cytotoxicity assays

As oncology drugs are generally cytotoxic, we wanted to identify the minimal concentrations of BCNU necessary for cytotoxicity. Neuro-2A (N2a) cells were incubated with BCNU at final concentrations of 0, 0.1, 1.0, 5.0, 10.0, 20.0, 80.0 and 240.0 uM for 24 hours. To determine cell viability, first we used the calorimetric 3-(4,5-dimethylthiazol-2-yl)-2,5-diphenyltetrazolium bromide (MTT) metabolic activity assay using a cell growth determination kit (cat # CGD-1, Sigma Aldrich) according to the manufacturer's instructions. Briefly, the supernatant was removed, cells were washed two times with PBS and 20 μl of MTT solution (5 mg/ml in PBS) plus 100 μl of medium were added. Following four hours of incubation at 37°C, the resulting formazan crystals were dissolved in 100 μl of DMSO and the absorbance was read at 570 nm within an hour using the Smart Spec Plus spectrophotometer (Bio-Rad). Cells treated with medium only served as controls.

To reproduce and confirm these results, we also measured cell viability using an *in vitro *toxicology assay kit based on secretion of lactic dehydrogenase (LDH) (cat # TOX7, Sigma Aldrich). The assay is based on reduction of nicotinamide adenine dinucleotide (NAD) by LDH enzyme into NADH which converts tetrazolium dye to a colored compound that can be quantitated spectrophotometrically. The procedure briefly is as follows: after 24 hours incubation of cells with BCNU at different concentrations, 50 μl of LDH assay lysis solution was added to each well and further incubated at 37°C for 45 minutes. The assay mixture was freshly prepared by adding equal volumes of substrate, dye and cofactor; 50 μl of LDH assay mixture was added to each of the 50 μl aliquots of the test medium. The plates were sealed with aluminum foil to protect from light and incubated for 30 minutes at 37°C. The reaction was terminated by adding 10 μl of 1 N HCl and the absorbance was measured at a wavelength of 490 nm.

### Staining of amyloid plaques

APdE9 mice that overexpress both APP with Swedish mutation and PS1 with ΔE9 deletion were used as a robust mouse model of Alzheimer's disease. All animal procedures were carried out strictly following the National Institutes of Health's 'Guide for the Care and Use of Animals' and using the animal protocol as approved (protocol # TPI-03-11) by the Torrey Pines Institute's Animal Care and Use Committee (IACUC). BCNU, dissolved initially in DMSO and further diluted in saline, was administered to mice daily by intraperitoneal injections at 0.5 mg/kg body weight starting from four months of age until six months of age for 60 days. Age and genotype-matched control mice received vehicle injections for the same period of time. Following the treatment period, mice were anesthetized by isoflurane and perfused using a mixture of 4% PFA and 0.02% glutaraldehyde in PBS. After 72 hours, the brains were dehydrated using sucrose gradient. Brains were frozen in optimal cutting temperature (OCT) solution and coronal sections of 16 μM thickness were cut by cryostat at -19°C to 21°C and transferred to superfrost slides. The slides were rinsed twice with distilled water for five minutes. The slides were then immersed in 1% thioflavin S solution prepared in 50% ethanol for five minutes and then differentiated in 70% ethanol for five minutes, rinsed again twice in water for five minutes and cover-slipped with Sure mount (EMS) mounting media and held at 4°C until they were imaged. Images were captured using a Zeiss Examiner D1 microscope. All images were acquired at the same exposure and were automatically aligned using the stitching tool in the Axiovision LE software. Once acquired, all images were opened in ImageJ and were normalized; the threshold was set for each image using the histogram mean at the same standard deviation. Each image was adjusted to the threshold and set to scale in pixels. The parameters measured include the area, integrated density, perimeter, and feret's diameter for each plaque. To help eliminate background the particle size pixel was set at 30-infinity pixel. To quantify plaques, the brain level of cut sections was fixed for all mice at the region of the motor cortex and hippocampus corresponding to the starting section at interaural 2.34 mm and Bregma -1.46 mm of 'The Mouse Brain Atlas' by George Paxinos and Keith Franklin. A fixed thickness of 16 μM coronal sections at regular intervals was maintained in all animals. The amyloid plaques were quantified from throughout the sections from five sections per mouse and mean values were generated for each mouse. Pictures were montaged and, for quantification by image J software, the color images were converted in to HSV format and 8-bit channels. Plaques were quantified in an unbiased manner by an investigator blind to the treatment nature of the samples. Plaque burden was calculated as the area occupied by the plaques divided by the total brain region area. The data are expressed as percent change in means from the controls.

### Quantitation of Aβ40 levels in the brain by ELISA

Aβ40 levels in the brain extracts were determined by sandwich ELISA. Briefly, the wet mass of the brain was weighed and homogenized thoroughly in cold 1% CHAPSO/PBS with protease inhibitors. The homogenate was ultra-centrifuged at 100,000 g for 60 minutes. The samples were further diluted to 40-fold and stored on ice until use. The Aβ standard (Bachem) was dissolved in hexafluoroisopropanol at 1 mg/ml, sonicated and dried under nitrogen. The dried Aβ40 was resuspended in DMSO, separated into aliquots and frozen at -80°C. The rest of the protocol is exactly as described previously from our laboratory [28]. The quantity of Aβ40 in each sample was measured in quadruplicate. Total protein concentrations were determined using the BCA assay (Pierce).

### Iba1 I mmunohistochemistry

Brain sections (18 um) from saline- and BCNU-treated mice were washed two times with PBS 1X for five minutes. Antigen retrieval was carried out by immersing slides in 10 mM citric acid (pH 6.0) for 10 minutes at 90°C. Sections were washed three times with PBS 1X for five minutes and incubated in blocking solution (10% normal goat serum, 1% BSA, 0.1% Triton X-100 in PBS 1X) for one hour at RT. The sections were incubated overnight with anti-Iba1/ALF1 mouse monoclonal antibody (Millipore) in blocking solution (1:200) at 4°C. After washing three times in PBS 1X for 5 minutes, the sections were incubated with Alexa Fluor^® ^568 goat anti-mouse IgG (Invitrogen) in blocking solution (1:500) at RT for two hours in the dark. Finally, slides were washed three times with PBS for five minutes, covered with mounting medium for fluorescence with 4',6-diamidino-2-phenylindole (DAPI) (Vector Laboratories) and sealed with nail clear. Sections were visualized in a fluorescence microscope (Axio Examiner D1) and a confocal microscope (Nikon 90i, scan head-C1 SHS, Melles Griot laser system). Microglia were counted in a defined area of the motor cortex and hippocampus (CA3) using the Image-Pro Plus (Media Cybernetics) software package. Positive cells were defined as those whose nuclei and processes were evidently stained for Iba1 and whose nuclei were co-localized with DAPI.

### Quantitation of BCNU drug levels in the brain

Two sets of three mice each were injected with BCNU at 4 mg/kg body weight and euthanized after either 5 or 20 minutes. The brains were rapidly removed, frozen and weighed before being homogenized in 250 μL of Dulbecco's PBS (dPBS), immediately followed by 750 μL of acetonitrile. The samples were spun for 10 minutes at 10,000 rpm at 4°C. The supernatant was removed and the samples were dried for two hours. The pellets were left at 4°C overnight, and on the following day samples were reconstituted in 30% acetonitrile, vortexed, and spun at 13,000 rpm for five minutes at 4°C. To assess stability of BCNU in the blood, blood samples were collected from several mice after anesthesia with isoflurane and 200 μL of blood was mixed with 8 μL of EDTA (final concentration 1.5 mg/mL) and about 100 μg of BCNU in 4 μl. The mixture was allowed to stand in a 37°C water bath for 0, 5, 10, 15 and 30 minutes. After the chase time, 600 μL of 100% acetonitrile was added to the sample. The samples were spun at 3,000 g for five minutes and the supernatant was collected. Each sample was made in triplicate, with the exception of the 0 minute blood which was done in duplicate. The blood samples were dried for approximately three hours. The pellet was reconstituted using 100 μL of 30% acetonitrile, vortexed and spun at 13,000 g for five minutes. The samples were transferred to shelf pack vials and run on the UV mass spectrometer. Similarly, to assess BCNU stability in the brain homogenates, three mice were anesthetized with isoflurane, perfused with dPBS and their brains collected. Brains were homogenized in 250 μL of dPBS and 200 μL of brain homogenate was incubated with 100 μg of BCNU for 1, 15, or 30 minutes at 37°C. After the chase time, 750 μL of 100% acetonitrile was added to the sample. The rest of the procedure was similar to the samples prepared for blood. All analyses were performed in triplicate.

The samples were then run on the UV mass spectrometer. The liquid chromatography system (Shimadzu, Kyoto, Japan) consisted of a LC20AD binary solvent delivery pump, a DGU-20A_5 _degasser, CTO-20A column oven and a SPD-M20A photodiode array detector. Chromatographic separation was carried out on a C-18 reverse phase column (Luna 50 μ, 100 Å, 50 × 4.6 mm) fitted with a C-18 reverse phase guard cartridge (Phenomenex, 4 × 3.00 mm) and BCNU was eluted using a gradient of solvents A (0.1% formic acid in water) and B (0.1% formic acid in acetonitrile) at 0.5 mL/minute flow rate. The gradient was 5% to 65% B over 20 minutes, 65% to 95% B over one minute, and kept for two minutes and restored to 5% B in one minute followed by re-equilibration for five minutes. The peak for BCNU was monitored at 280 nm by injecting 5 μL of the sample.

### Statistical analysis

The signal intensities of immunoblots of the samples treated with BCNU in CHO cells as well as plaque burden in the mouse brain were quantified using publicly available Java-based ImageJ software developed at the National Institutes of Health. All data were analyzed by Student's t test using Instat3 software (GraphPad Software, San Diego, CA, USA). We used a two-tailed *P *value assuming populations may have different standard errors. The data for dose-response experiments were analyzed by analysis of variance (ANOVA) followed by either Dunnett or Bonferroni multiple comparison tests. The data were considered significant only if the *P *< 0.05, * indicates *P *< 0.05, **, *P *< 0.01 and ***, *P *< 0.001.

## Results

### BCNU decreases Aβ levels dose-dependently in CHO cells

**Figure 1:**
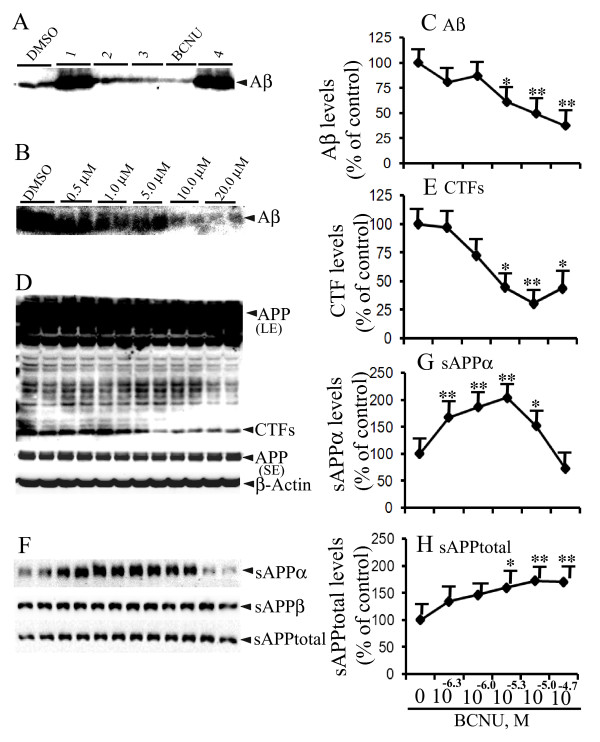
**BCNU decreases Aβ and CTF levels in CHO cells stably expressing APP751WT (7WD10 cells)**. **A**) An initial screening of a library of oncology drugs used at 10.0 μM identified carmustine (BCNU) as a potential anti-Aβ drug, while drugs such as 1 and 4 increased Aβ levels. The cells were exposed to the drugs for 48 hours and the conditioned media were immunoprecipitated for Aβ and detected by immunoblots. **B**) 7WD10 cells were incubated with different concentrations of BCNU for 48 hours and the conditioned media were immunoprecipitated for Aβ and detected by immunoblots. **C**) Quantitation of Aβ levels by ImageJ revealed decreased Aβ levels to 61% (*P *< 0.05) at 5 μM, 49% (*P *< 0.01) at 10 μM and 37% (*P *< 0.01) at 20 μM compared to controls. **D) **The lysates were used to detect c-terminal fragments (CTFs) and APP as well as actin as a loading control. **E**) CTF levels were also significantly decreased to 44% (*P *< 0.05) at 5 μM, 30% (*P *< 0.01) at 10 μM and 43% (*P *< 0.05) at 20 μM compared to controls. **F**) The conditioned media from B was subjected to immunoblotting to detect different species of sAPPs. **G**) sAPPα levels were increased to 167% (*P *< 0.01) at 0.5 uM, 186% (*P *< 0.05) at 1 uM, 204% (*P *< 0.01) at 5 uM and 152% (*P *< 0.05) at 10 uM compared to untreated cells. **H**) sAPPtotal levels were increased to 159% (*P *< 0.05) at 5 μM, 172% (*P *< 0.01) at 10 μM and 170% (*P *< 0.01) at 20 μM in BCNU treated cells compared to controls. For all samples, n = 4 ± SEM. *, *P *< 0.05, **, *P *< 0.01 versus control by analysis of variance (ANOVA) followed by post-hoc test by Dunnett multiple comparisons. Aβ, amyloid β; APP, Alzheimer's precursor protein; BCNU, 1, 3 bis (2-chloroethyl)-1-nitrosourea; CHO, Chinese hamster ovary; SEM, standard error of the mean.

Anecdotal observations in nursing homes that cancer survivors were less likely to be diagnosed with AD which was confirmed in multiple studies [25,26,30] strongly suggest an inverse relationship between cancer and AD. This compelling evidence led us to firmly believe that oncology drugs might be helpful in AD. Therefore, we screened a library of all the FDA-approved oncology drugs totaling 89 compounds obtained from NCI/NIH [27] at a concentration of 10.0 μM to determine their effects on Aβ levels by immunoprecipitation of Aβ in the CM and Western blotting. Interestingly, BCNU strongly decreased Aβ levels in CHO cells in the initial screens (Figure 1A). Subsequent dose-response experiments confirmed that BCNU induced decreased Aβ levels in CHO cells (Figure 1B &1C). To test the effect of different concentrations of BCNU, CHO cells stably expressing APP751WT (7WD10) were treated for 48 hours and the CM were immunoprecipitated with Ab9 antibody which recognizes an epitope within 1-16 amino acids of Aβ peptide. This was followed by a Western blot detection of Aβ using the 6E10/82E1 mixture of antibodies which we have previously used for consistent detection of total Aβ species [28,29]. Exposure of 7WD10 cells to BCNU decreased the secretion of Aβ starting at 5.0 μM by 39% (*P *< 0.05), 10.0 μM by 51% (*P *< 0.01) and 20 μM by 63% (*P *< 0.01) compared to cells treated with a structural analog as controls (Figure 1B and 1C). Thus, increasing the concentration of BCNU revealed a dose-dependent decrease in Aβ levels, without altering the level of the holoprotein at any of the concentrations tested. These results demonstrate that BCNU inhibits β/γ-secretase mediated APP cleavage of WT APP.

In an effort to identify more potent carmustine analogs, we synthesized 12 carmustine structural derivatives and screened them for their effect on Aβ levels. The derivatives tested include 1-(2-chloroethyl)-3-hexylimidazolidin-2-one (C_11_H_21_ClN_2_0; MW, 232.7502), 1-(2-chloroethyl)-3-(3-isopropylphenylimidazolidin-2-one (C_14_H_19_ClN_2_0; MW, 266.7665), 1, 3-bis(2-chloroethylimidazolidin-2-one (C_7_H12Cl_2_N_2_0; MW, 232.7502), 1-(2-chloroethyl)-3-(3-phenylimidazolidin-2-one (C_11_H_13_ClN_2_0; MW, 224.6867), 1-(2-bromoethyl)-3-(2-chloroethylimidazolidin-2-one (C_7_H_12_BrClN_2_0; MW, 255.5400), 1-(2-chloroacetyl)-3-(2-chloroethyllimidazolidin-2-one (C_7_H_10_Cl_2_N_2_0_2_; MW, 225.0725), 1-(2-chloroethyl)-3-phenylimidazolidin-2-thione (C_11_H_13_ClN_2_S; MW, 240.7523), phenyl ethylamine (C_19_H_25_N_3_O; MW, 311.20), cyclopentyl amine (C_13_H_25_N_3_O; MW, 239.20), butyl amine (C_11_H_25_N_3_O; MW, 215.20), piperidine (C_13_H_25_N_3_O; MW, 239.20) and morpholine (C_11_H_21_N_3_O_3_; MW, 243.16). None of the derivatives were as potent as BCNU.

### BCNU decreases CTF levels dose-dependently in CHO cells

To quantify APP-derived CTF levels, lysates were prepared from the same cells that were used for Aβ quantitation after treatment with different concentrations of BCNU. CTFs were detected by Western blotting using CT15 antibody. Similar to Aβ levels, CTF levels were also significantly decreased at 5.0 μM (55%, *P *< 0.05), 10.0 μM (70%, *P *< 0.01) and 20.0 μM (56%, *P *< 0.05) compared to untreated controls (Figure 1D and 1E). Thus, except for the first two lower concentrations, BCNU significantly decreased APP processing and CTF production, starting from 5.0 μM. These changes occurred without alterations in APP holoprotein which was also detected by the CT15 antibody. The decreased CTF levels are consistent with decreased Aβ levels by BCNU and indicate that BCNU decreases amyloidogenic processing of APP in CHO cells.

### BCNU significantly increases levels of sAPPα and sAPPtotal but not sAPPβ

To obtain an overall picture of APP metabolism under BCNU treatment, we further quantified the levels of secreted large extracellular N-terminal domain truncated protein at the α-site (sAPPα) or at the β-site (sAPPβ) as well as levels of sAPPtotal from the same CM used for Aβ quantification. More than a one-fold increase was noted for sAPPα levels at some concentrations of BCNU applied for 48 hours, although we did not notice a strict dose-dependent increase in sAPPα levels. The secretion of sAPPα was increased to 167% at 0.5 μM (*P *< 0.01), 186% (*P *< 0.05) at 1.0 μM, 204% (*P *< 0.01) at 5.0 μM and 152% (*P *< 0.05) at 10.0 μM (Figure 1F and 1G). For some unknown reason, sAPPα levels were not altered at the highest concentration of 20.0 μM tested, although Aβ levels were significantly decreased. The maximum increase (204%) was noted at 5.0 μM concentration. Similarly, the levels of sAPPtotal were increased by 159% (P < 0.05) at 5.0 μM, 172% (*P *< 0.01) at 10.0 μM and 170% (*P *< 0.01) at 20.0 μM in BCNU-treated cells (Figure 1F and 1H). On the other hand, there were no significant alterations in the levels of sAPPβ at any of the BCNU concentrations tested. The sAPPα levels were detected using 6E10 antibody which recognizes an epitope within the 1-17 amino acid of the Aβ domain of APP. sAPPtotal was detected by the 63G antibody whose epitope lies within the mid region of APP. Taken together, decreased levels of Aβ and CTFs and increased release of sAPPα and sAPPtotal in the CM clearly indicate that BCNU may decrease amyloidogenic processing of APP by increasing α-secretase mediated cleavage of APP thereby reducing the APP substrate available for cleavage by BACE enzyme.

### BCNU increases immature APP at the cell surface

**Figure 2:**
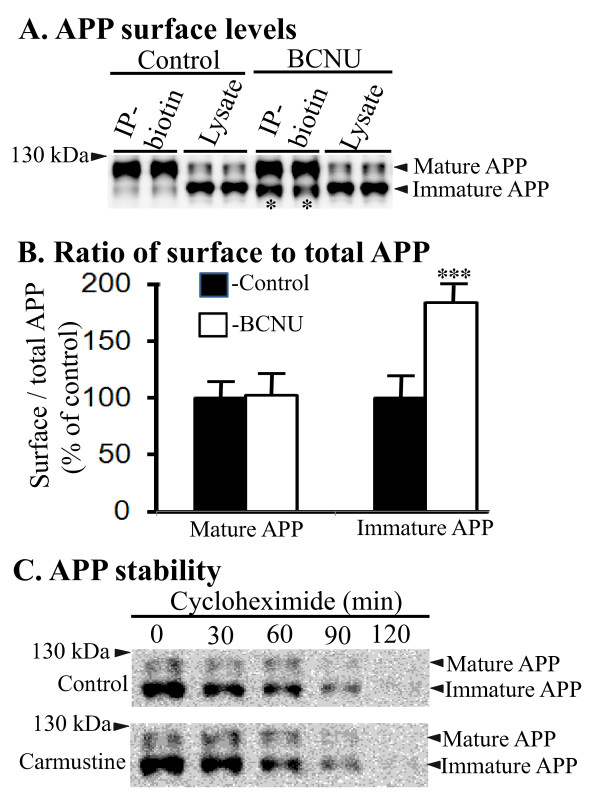
**BCNU increases surface levels of immature APP**. **A**) Surface biotinylation and biotin immunoprecipitation experiments showed significantly increased surface levels of immature APP in BCNU-treated 7WD10 cells compared to untreated cells. **B**) Quantitation of the signal in A) revealed increased surface to total APP levels for immature APP by 184% but the levels of mature APP were unaltered. **C**) Treatment of 7WD10 cells with BCNU at 10.0 uM concentration followed by cycloheximide chase at indicated time points did not alter APP half-life or stability. For all samples, n = 4 ± SEM. ***, *P *< 0.001 versus controls by t test. APP, Alzheimer's precursor protein; BCNU, 1, 3 bis (2-chloroethyl)-1-nitrosourea; SEM, standard error of the mean.

As endocytosis of surface APP is required for Aβ generation [31,32], we next examined whether BCNU treatment altered surface levels of APP. To do this, all surface proteins were labeled with biotin and immunoprecipitated with antibiotin antibody. Results showed that BCNU-treated cells accumulated immature APP by more than one-fold at the cell surface compared to untreated cells (*P *< 0.001), while there was no change in the surface levels of mature APP (Figure 2A and 2B). Next, to test whether decreased amyloidogenic processing of APP is due to changes in turn-over of APP, we performed cycloheximide chase experiments. Confluent 7WD10 cells in six-well plates treated with or without BCNU were incubated with 100 mg/ml of cycloheximide to inhibit *de novo *synthesis of APP. This was followed by quantitation of steady state levels of APP holoprotein by Western blots at different time points up to two hours based on our previous experience that the calculated half-life of APP was about one hour [28]. The results did not reveal significant differences in the levels of APP at any of the time points tested between untreated and BCNU-treated cells (Figure 2C). This suggests that BCNU does not affect the half-life of APP and its stability. The decreased levels of Aβ and CTFs induced by BCNU treatment, therefore, might result from increased immature APP at the cell surface leading to reduced endocytosis of APP which is necessary for cleavage of APP by secretases for Aβ production [31,32].

### BCNU does not inhibit secretases

**Figure 3:**
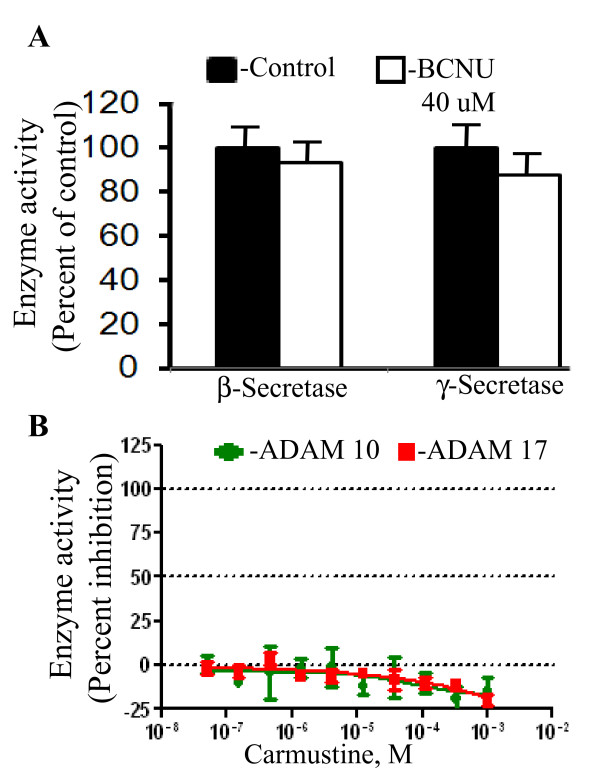
**BCNU does not alter the activities of secretases**. **A**) Partially purified β- and γ-secretase enzyme complexes from mouse brain homogenates were used to detect the activity of secretases using specific fluorogenic substrates. BCNU up to 40 μM did not inhibit the activities of either β- or γ-secretases. **B**) The activities of both ADAM17 and ADAM10 (α-secretase) were also not affected by BCNU at any of the concentrations tested. BCNU, 1, 3 bis (2-chloroethyl)-1-nitrosourea.

APP is cleaved by sequential actions of β- and γ-secretases to release Aβ and, therefore, compounds that reduce Aβ generation can be expected to inhibit secretases. Therefore, we tested whether BCNU inhibits any of the secretases. To our surprise the activities of both β- and γ-secretases were not affected by BCNU even at concentrations as high as 40 μM of BCNU (Figure 3A). Similarly, we did not notice any change in the activities of ADAM 17 or ADAM 10 (Figure 3B), which cleaves APP at the α-site. Thus, BCNU appears to decrease Aβ generation independent of secretases, probably by simply altering the trafficking of APP.

### Cytotoxicity of BCNU measured by LDH release and MTT reduction

**Figure 4:**
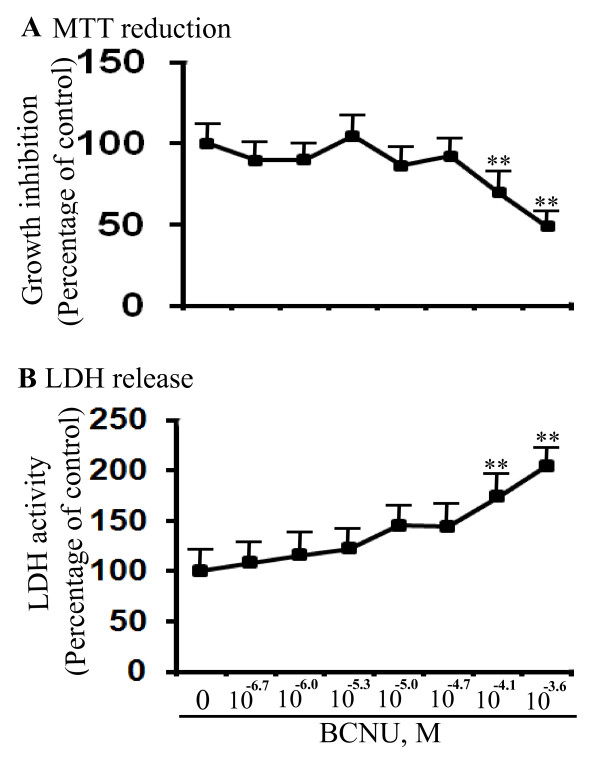
**BCNU is cytotoxic only at high concentrations in Neuro-2a (N2a) cells**. **A**) LDH release was measured as an indicator of cytotoxicity after incubation with different concentrations of BCNU as indicated. BCNU was not toxic up to 20 μM and significant cytotoxicity was observed only at 80 μM (*P *< 0.01), and120 μM (*P *< 0.01). **B**) The MTT reduction assay also indicated significant toxicity only at 80 μM (69%, *P *< 0.01) and 240 μM (48.7%, *P *< 0.001) but BCNU was nontoxic up to 20 μM. For all samples, n = 4 ± SEM. **, *P *< 0.01versus untreated controls by analysis of variance (ANOVA) followed by post-hoc test by Dunnett multiple comparisons. BCNU, 1, 3 bis (2-chloroethyl)-1-nitrosourea; LDH, lactate dehydrogenase; MTT, 3-(4, 5-dimethylthiazol-2-yl)-2, 5-diphenyltetrazolium bromide.

The cytotoxicity of BCNU was determined by two independent enzyme-based assays by colorimetric detection using neuron-derived neuro-2a (N2a) cells. The LDH release assay revealed BCNU was nontoxic up to 20 μM and was toxic only at 80 μM (69.5%, *P *< 0.01) and 240 μM (49%, *P *< 0.01) (Figure 4B). To confirm these results by another method, we used CM from the cells incubated with different concentrations of BCNU to quantify the reduction of the MTT substrate. The MTT reduction assay reproduced the results of the LDH assay in that BCNU was non-toxic up to a concentration of 20 μM. However, BCNU incubation at 80 μM (50%, *P *< 0.01) and 240 μM (45%, *P *< 0.01) was significantly toxic at each concentration (Figure 4A). Thus, it is interesting to note that BCNU is toxic to the neuronal cell line only at high concentrations but not at concentrations that decreased Aβ levels.

### Chronic BCNU administration decreases plaque burden in mice

**Figure 5:**
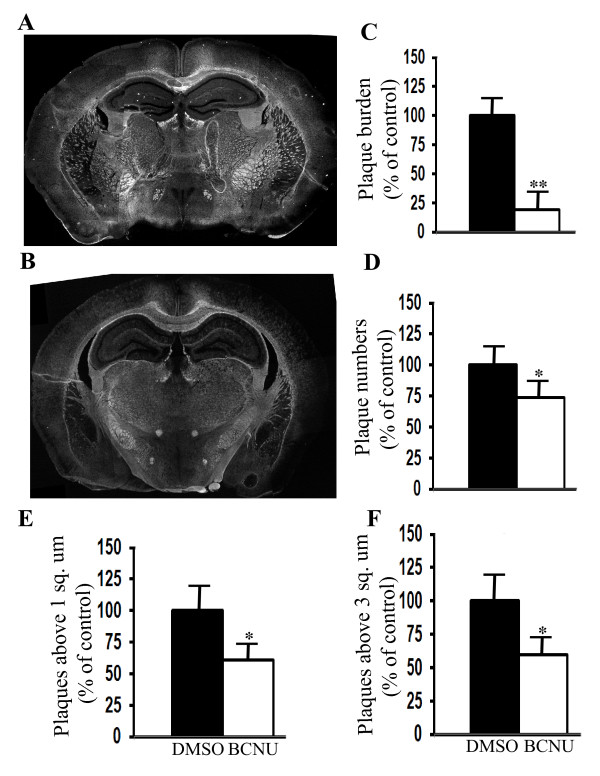
**Chronic BCNU administration decreases amyloid plaque burden in mice**. APdE9 mice were treated daily by intraperitoneal injections with BCNU starting from four months to six months of age for 60 days. Amyloid plaques were stained by thioflavin S in coronal brain sections. **A**) A representative section with amyloid plaques in vehicle-treated mice at six months of age is shown. **B**) A representative section of brain showing decreased amyloid plaques in mice treated with BCNU is shown. **C**) Quantitation by ImageJ analysis showed decreased plaque burden by 81% (*P *< 0.01) when compared to vehicle-treated APdE9 mice. **D**) The total number of plaques was reduced by 26% (*P *< 0.05). **E**) Plaques measuring more than 1.0 square micron were reduced by 39% (*P *< 0.05). **F**) Plaques measuring more than 3.0 square microns decreased by 41% (*P *< 0.05). In each group, n = 6, ± SEM. *, *P *< 0.05, **, *P *< 0.01 in BCNU-treated APdE9 mice versus vehicle-treated APdE9 mice by student's t test. BCNU, 1, 3 bis (2-chloroethyl)-1-nitrosourea; SEM, standard error of the mean.

Although BCNU decreased Aβ production in cell cultures, amyloid plaques in the brain are more relevant to neurodegeneration in AD. Therefore, to verify whether decreased amyloidogenic processing of APP by BCNU observed in cell cultures is translated *in vivo *into decreased plaque burden, mice overexpressing APP with Swedish mutation and PS1 with ΔE9 deletion were used as a robust mouse model for Aβ plaques (APdE9) as these mice develop a modest amount of amyloid plaques as early as six months of age. Overall plaque burden was calculated as the ratio of 'the area occupied by plaques to the total region area', which was clearly decreased by about 81% (*P *< 0.01) in BCNU-treated mice compared to saline-treated mice (Figure 5A, B and 5C). A representative histology section for saline- or BCNU-treated mice is shown in Figure 5A and 5B at comparable brain levels. The total plaque numbers were reduced only by 26% (Figure 5D, *P *< 0.05). However, when the plaques were counted based on their size, those plaques which measured more than 1.0 square micron decreased by 39% (Figure 5E, *P *< 0.05) and those measuring above 3.0 square microns decreased by 41% (Figure 5F, *P *< 0.05) in the BCNU-treated mice compared to the saline-treated mice. Highly decreased plaque burden and a modest reduction in plaque numbers in BCNU-treated mice indicates that BCNU decreases the severity of plaques by reducing larger plaques. The decreased amyloid plaque burden following chronic BCNU administration in mice is consistent with decreased amyloidogenic processing of APP and Aβ levels observed in cell cultures (Figure 1A, B and 1C).

### BCNU decreases levels of Aβ40 and CTFs and increases sAPPα in mouse brains

**Figure 6:**
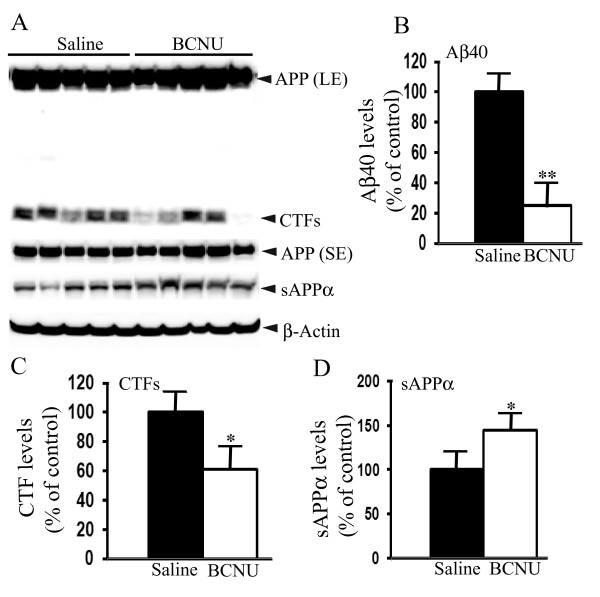
**Chronic BCNU administration alters APP metabolites in the mouse brain**. Mice were treated as in legends to Figure 5. **A**) Immunoblots showing APP (long exposure, LE and short exposure, SE), CTFs, sAPPα and actin as loading control. **B**) Quantification of Aβ by sandwich ELISA revealed robust reduction of Aβ40 by 75% (*P *< 0.01) in BCNU-treated brains compared to saline-treated mice. **C**) Quantification of CTFs also revealed significant reductions by 39% (*P *< 0.05) in BCNU-treated mouse brains. **D**) sAPPα levels were increased by 45% (*P *< 0.05) in BCNU-treated mice. In each group, n = 5, ± SEM. *, *P *< 0.05, **, *P *< 0.01 in BCNU-treated APdE9 mice versus vehicle-treated APdE9 mice by student's t test. Aβ, amyloid β; APP, Alzheimer's precursor protein; BCNU, 1, 3 bis (2-chloroethyl)-1-nitrosourea; CTFs, c-terminal fragments; SEM, standard error of the mean.

To test whether the decrease in amyloid plaques by BCNU was due to a decrease in Aβ, we quantified the levels of Aβ40 in the mouse brains by sandwich ELISA using Aβ specific antibodies. Exposure of BCNU even just for two months resulted in a 75% reduction in the levels of Aβ40 (Figure 6B), which correlates well with the amount of reduction in amyloid plaques. Similar to the effect in cell cultures, CTF levels were also reduced in the brains after BCNU treatment by 39% (*P *< 0.01) (Figure 6C). Conversely, the levels of sAPPα were increased by 45% (*P *< 0.01) (Figure 6D). Thus, BCNU-induced changes in APP metabolites observed in cell cultures were confirmed *in vivo *in the mouse brains.

### BCNU is rapidly metabolized in mice

**Figure 7:**
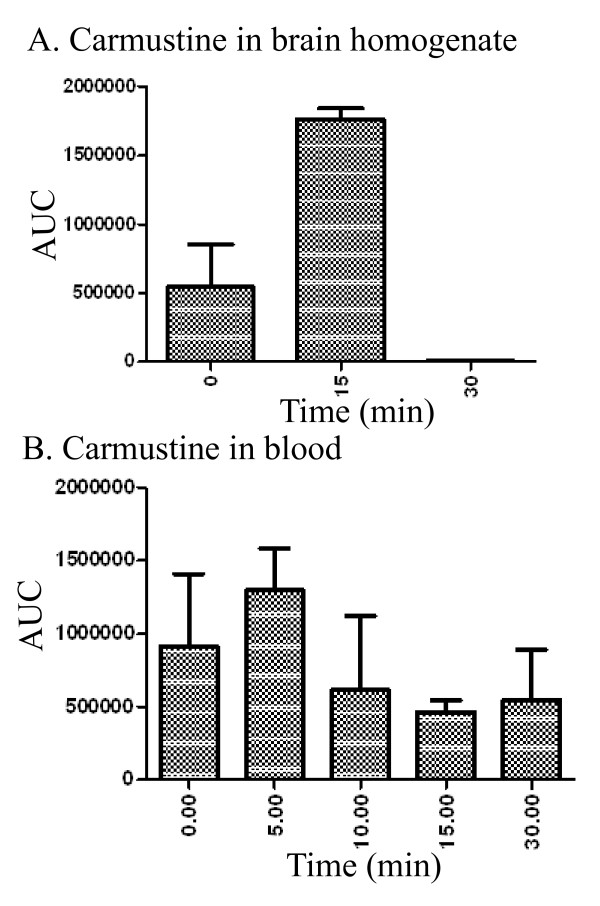
**BCNU is relatively unstable in tissues**. **A**) Incubation of BCNU in mouse brain homogenates showed intact BCNU only up to 15 minutes, but at 30 minutes, there was no trace of BCNU. **B**) Intact BCNU, however, could be detected in the blood for up to 30 minutes. BCNU, 1, 3 bis (2-chloroethyl)-1-nitrosourea.

**Figure 8:**
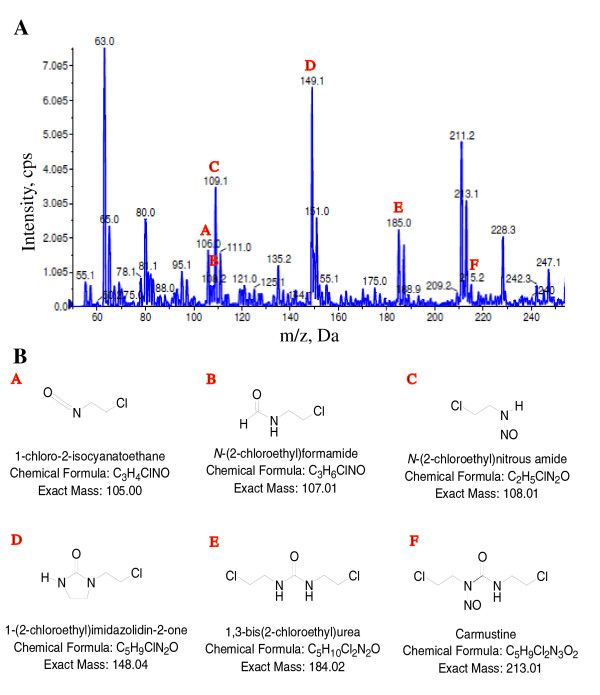
**Chloro isotopic distribution of the main metabolites identified in the brain homogenates after BCNU administration**. **A) **Chromatogram showing distribution of BCNU metabolites, labeled A to F with their molecular weights. **B**) Chemical structures of the identified metabolites, A to F. BCNU, 1, 3 bis (2-chloroethyl)-1-nitrosourea.

In order to correlate the drug concentrations in the brain that are required to reduce amyloidogenic processing of APP and plaque burden, we quantified BCNU levels in the brain by liquid chromatography with UV detection. At least three mice were tested for each of 5 and 20 minute time points following tail vein injection. Surprisingly, we failed to detect any BCNU at either time point. Direct incubation of BCNU in the blood and brain homogenates confirmed that BCNU was rapidly degraded with no trace of the compound in the brain homogenates within 30 minutes (Figure 7A), although BCNU could be detected up to 30 minutes in the blood (Figure 7B). These results suggest that BCNU is rapidly metabolized and the biological effect exerted on Aβ levels could result from the action of one of the metabolites. In fact, the distribution and clearance of BCNU in both human and animals have been extensively studied. BCNU is a very unstable compound which undergoes both *in vitro *and *in vivo *spontaneous degradation to isocyanate and the chloroethylodiazohydroxide ion, which then is degraded to the chloroethylocarbonic ion and others [33,34]. Careful analysis of the chromatograms of brain homogenates revealed the presence of several metabolites, labeled A to E, which we could identify in addition to several others which we could not identify (Figure 8A). We detected only a very small peak for the parent compound, BCNU, labeled F in the chromatogram (Figure 8A). The names and chemical structures of identified metabolites with their molecular weights are shown in Figure 8B. Such metabolites might actually be responsible for decreasing Aβ levels and plaque burden.

### BCNU suppresses microglial activation

**Figure 9:**
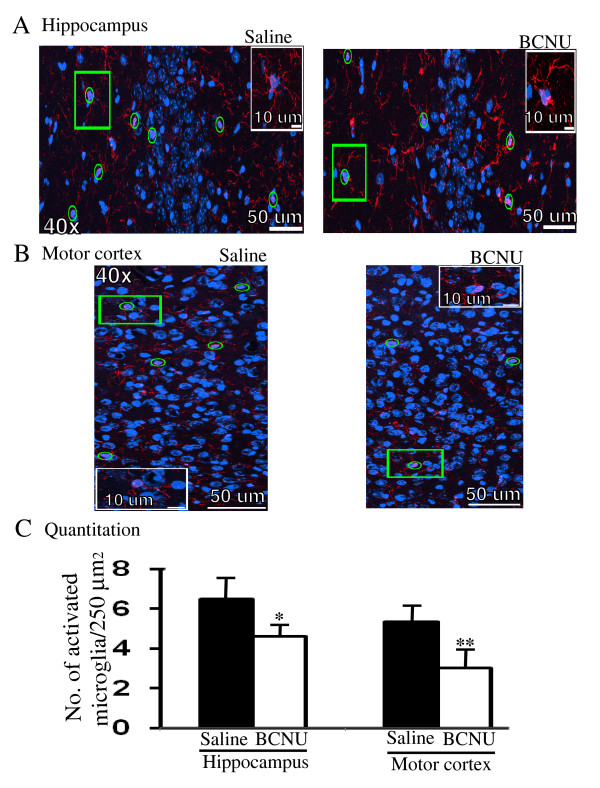
**The number of activated microglia is reduced in both the hippocampus and motor cortex in BCNU-treated mouse brains**. **A**) Iba1 positive activated microglia (red) in the CA3 region of the hippocampus shown in saline- or BCNU-treated mouse brains. The nuclei were stained with DAPI (blue) and the merged images (yellow) show microglia positive for Iba1, which were counted. **B**) Iba1 positive activated microglia in the motor cortex. The magnification and the scale bars are indicated. The inset in both A and B is to show an enlarged microglia positive for both DAPI and Iba1. **C**) Quantification showed 30% (*P *< 0.05) and 44% (*P *< 0.01) reduced numbers of activated microglia in the hippocampus and motor cortex, respectively. All values are mean ± SEM, n = 5 in each group. *, *P *< 0.05, **, *P *< 0.01 in BCNU-treated APdE9 mice versus saline-treated APdE9 mice by student's t test. BCNU, 1, 3 bis (2-chloroethyl)-1-nitrosourea; DAPI, 4',6-diamidino-2-phenylindole; SEM, standard error of the mean.

In order to understand whether BCNU-mediated decreased plaque burden results from an increased number of activated microglia as previously shown for some oncology drugs, we quantified the number of activated microglia by staining brain sections with anti-Iba1 antibody. On the contrary, as shown in Figure 9A and 9C, chronic BCNU treatment resulted in a lower number of Iba1-positive microglia in the hippocampus (30%, *P *< 0.05). Similarly we observed a 44% (*P *< 0.01) lower number of Iba1-positve microglia in the motor cortex of BCNU-treated mouse brains as compared to saline-treated controls (Figure 9B and 9C). Thus, BCNU, in contrast to other oncology drugs, reduces microglial activation. This may even have a positive effect on the brain since activated microglia is pro-inflammatory and may lead to neurodegeneration.

### BCNU increases transforming growth factor β levels

**Figure 10:**
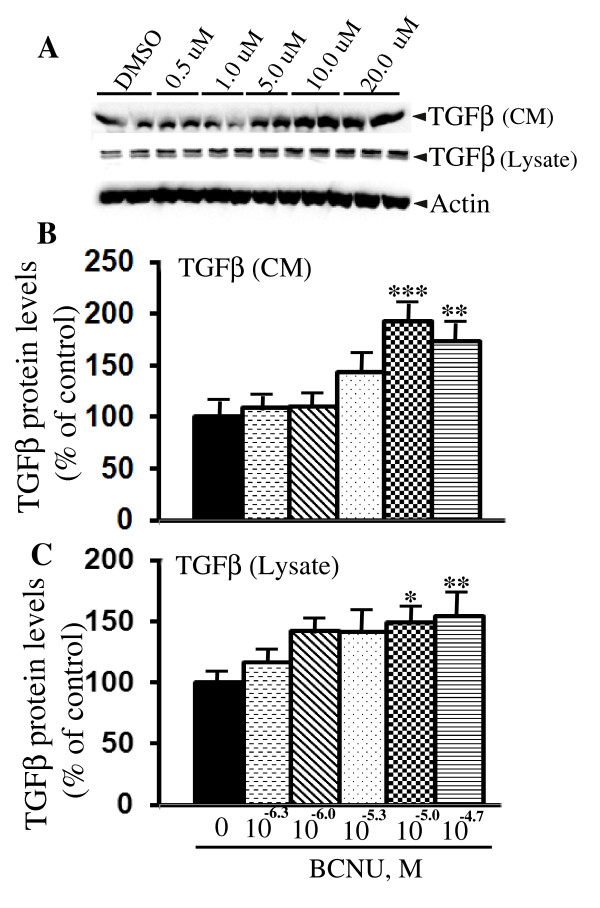
**BCNU increases TGFβ protein levels in 7WD10 cells**. **A**) Cells were treated with BCNU at different concentrations as indicated and, after 48 hours, the conditioned media (CM) as well as lysates were immunoblotted to detect TGFβ protein. **B**) ImageJ quantitation revealed significantly increased TGFβ protein levels at 10.0 μM (73%, *P *< 0.01) and 20.0 μM (92%, *P *< 0.001) in the CM. Similarly in the lysates TGFβ levels were increased at the same concentrations of 10.0 μM (149%, *P *< 0.05) and 20.0 μM (154%, *P *< 0.01). For all samples, n = 4 ± SEM. *, *P *< 0.05, **, *P *< 0.01, ***, *P *< 0.001versus untreated controls by analysis of variance (ANOVA) followed by post-hoc test by Dunnett multiple comparisons. BCNU, 1, 3 bis (2-chloroethyl)-1-nitrosourea; SEM, standard error of the mean; TGFβ, transforming growth factor β.

In a study using mRNA differential display, BCNU has been shown to increase the expression of a gene encoding the latent transforming growth factor-binding protein 1 (LTBP-1) by three-fold suggesting that BCNU might increase transforming growth factor-β1 (TGF-β1) signaling [35], which is known to play a pivotal role in APP metabolism. Therefore, to understand the possible molecular mechanism for the reduced amyloidogenic processing of APP by BCNU, we quantified the levels of TGFβ protein released into the CM and also in the lysates after cells were treated with different concentrations of BCNU. Interestingly, TGFβ levels were increased at 10.0 μM (92%, *P *< 0.001) and 20.0 μM (73%, *P *< 0.01) compared to untreated cells (Figure 10A and 10B). TGFβ levels were also significantly increased in the lysates at the same concentrations of 10.0 μM (149%, *P *< 0.05) and 20.0 μM (154%, P < 0.01). The increased levels of TGFβ at the higher concentrations of BCNU are consistent with decreased Aβ levels at these concentrations.

## Discussion

In this study, we have shown for the first time that BCNU treatment can reduce amyloidogenic processing of APP and Aβ generation in cell cultures as well as in a mouse model of AD. Although BCNU is known to be a cytotoxic compound, it reduced Aβ levels at non-toxic concentrations. Two other oncology drugs have recently been shown to significantly reduce Aβ generation. Imatinib (Gleevec) has been shown to reduce Aβ by 50% at 10.0 μM concentration [36,37], similar to the magnitude observed for BCNU in the present study, but the advantage of BCNU is that it can pass through the BBB [38], while Gleevec cannot [39]. Bexarotene, another oncology drug, reduced amyloid plaques by 50% within 72 hours after oral administration [40]. Although BCNU reduced plaque burden by 81%, much more than did bexarotene, it took two months of intraperitoneal administration unlike bexarotene which rapidly cleared within a few days of oral treatment. Thus, BCNU falls between Gleevec and bexarotene as a favorable Aβ reducing drug.

It is noteworthy that BCNU decreased Aβ levels from CHO cells stably expressing APP751WT (7WD10 cells), as the majority of cases of sporadic AD patients carry wild-type APP. Thus, BCNU decreased Aβ generation from both the predominantly neuronal form, APP695, as well as the non-neuronal form, APP751, considering the results in 7WD10 cells as well as mouse brains. Wild-type APP and APP with Swedish mutation are known to be processed and trafficked differently. The most important observation that we made was the increased ratio of mature APP versus immature APP at the cell surface. The molecular mechanisms that regulate APP maturity, trafficking and Aβ generation are complex. Newly synthesized immature APP in the endoplasmic reticulum (ER) is first N-glycosylated at the ER and after its exit undergoes O-glycosylation at the Golgi complex attaining maturity. In the secretory pathway mature APP is then sorted to the plasma membrane where it undergoes endocytosis, a necessary step for Aβ production [31,32]. Thus, BCNU reduces Aβ generation probably by reducing endocytosis of APP from the cell surface. But Aβ can also be produced in the secretory pathway. If APP undergoes α-cleavage presumably in the secretory pathway in the trans-Golgi network, it can drastically reduce Aβ generation. Increased sAPPα levels in the present study also suggests that BCNU might influence Aβ generation through increased α-secretase mediated processing of APP. Decreased Aβ levels induced by BCNU may be a cumulative effect of both pathways. Interestingly, several pharmacological agents have been shown to reduce Aβ generation by attenuating APP maturity. For example, protein kinase A (PKA) inhibitors have been shown to reduce Aβ production by accumulating immature APP [41]. Inhibitors of acyl-coenzyme A cholesterol acyl transferase (ACAT) also reduced Aβ production by decreasing the ratio of mature to immature APP [42]. Similarly, zinc [43] and O-glycosylation inhibitors [44], both of which retarded APP maturation, also reduced Aβ generation. Since BCNU did not affect the activities of any of the secretases, reduced Aβ generation most likely results from altered trafficking of APP due to accumulation of immature APP. Protein overexpression studies also provide indirect evidence that maturity and trafficking of APP to the cell membrane affects Aβ production. Overexpression of growth arrest-specific 1 gene also reduces Aβ generation by inhibiting glycosylation of APP thereby enhancing accumulation of immature APP in the plasma membrane [45]. Collectively, these data provide compelling evidence that BCNU decreases Aβ production by retarding APP maturation thereby altering its trafficking and cleavage. It is also possible that BCNU decreased Aβ levels through the TGFβ pathway. Genetic polymorphisms at +10 CC genotype on TGFβ has been shown to be associated with reduced serum levels of TGFβ in mild cognitive impairment (MCI) patients later diagnosed as AD [46], and to increase the risk of developing late-onset AD [47]. Overexpression of TGFβ in the transgenic mouse models also reduces Aβ generation and plaque burden [48]. Thus, increased TGFβ levels in both the CM and lysates by BCNU are consistent with these data. The mechanism by which BCNU reduces Aβ and plaque burden might be mainly through its effect on intracellular trafficking and maturation of APP. Also, unlike other oncology drugs, BCNU reduced the number of microglia, ruling out the possibility that microglia are responsible for clearing amyloid plaques. One limitation of our study is that we did not co-stain microglia with an anti-Aβ antibody to stain amyloid plaques. The reduced number of microglia may be related to the decreased amyloid load. Since we did not co-stain, we cannot conclude that decreased microglia correlate with decreased plaque burden. Another limitation of our study is that we did not study the effect of short-term drug exposure on either amyloid plaques or microglia. Thus, after short-term drug treatment microglia numbers may be increased, but we do not have data to support this idea.

In cancer patients, BCNU is administered at 200 mg/m^2 ^every six weeks and since the body surface area in humans is 1.6, the total dose administered is about 320 mg/person (or approximately 4 mg/kg body weight), an equivalent of 300 μM in an adult. The major limiting factor of BCNU is its toxicity, particularly hepatic and pulmonary fibrosis. Therefore, to avoid systemic toxicity, patients with solid brain tumors are implanted with BCNU wafers, up to eight per patient, each with 7.7 mg of BCNU. Thus, a very high concentration of BCNU is released in the brain, which may be responsible for some of the adverse effects, such as the seizures and brain edema seen in patients implanted with BCNU wafers. Comparing these doses in humans and extrapolating to mice, our *in vivo *dose of 0.5 mg/kg body weight in mice, an equivalent of 30 μM is about 10-fold less compared to the dose in humans and, so, not expected to cause any systemic or neuro-toxicity. In fact we did not notice any signs of toxicity during the entire treatment period. However, in mice significant toxicity of BCNU in terms of increased cell death has been reported when BCNU is administered at 20 [49] or 10 [50] mg/kg body weight. Because the LD50 dose in mice for BCNU is 52 (males) and 46 (females) mg/kg body weight [51], respectively, our dose of 0.5 mg/kg is about 100 times lower than the LD50 dose in mice or 20 to 40 times lower than the doses used by other studies where significant toxicity was encountered. Taken together our data clearly suggest that BCNU reduces the levels of Aβ40 as determined using Aβ-specific antibodies and amyloid plaque burden determined by thioflavin S staining at non-toxic concentrations. Thus, this is definitive and confirmatory evidence that BCNU treatment reduces amyloidogenic processing of APP *in vivo*.

The most interesting observation made in the present study is that BCNU reduced amyloidogenic processing of APP independent of secretases. Thus, the plethora of side effects resulting from inhibition of secretases which is the predominant reason for their failures in clinics can be completely avoided [52,53]. The recent discovery of a coding mutation on the APP gene (A673T) which provides significant protection against cognitive decline in both AD patients as well as normal elderly individuals [54] is strong proof of the principle evidence that reducing Aβ levels is an effective therapeutic approach for AD. Therefore, discovery of any anti-Aβ drug, especially those which modulate Aβ generation independent of secretases would have wider clinical applications. Our finding that BCNU has a potent anti-Aβ effect is in line with the anecdotal clinical observations that AD and cancer are inversely related. Although there is no epidemiological evidence linking decreased risk of developing AD after chemotherapy, our data suggest that cancer drugs, such as BCNU, can be effective therapeutic agents against AD.

## Conclusions

In summary, our data have revealed for the first time that chronic BCNU administration at a non-toxic dose leads to robust reduction of Aβ40 levels and amyloid plaque burden as a consequence of decreased amyloidogenic processing of APP. Decreased amyloid plaques may possibly result from altered trafficking and processing of APP. Thus, BCNU may emerge as a novel and powerful anti-Aβ drug for the effective treatment and prevention of AD. Since BCNU acts independent of secretases, all the inadvertent side effects due to their off-target effects can be completely avoided.

## Abbreviations

Aβ: amyloid β; ACAT: acyl-coenzyme A cholesterol acyl transferase; AD: Alzheimer's disease; APP: amyloid precursor protein; BACE: β-secretase; BBB: blood brain barrier; BCNU: 1, 3 bis (2-chloroethyl)-1-nitrosourea; BSA: bovine serum albumin; CAA: cerebral amyloid angiopathy; CHO cells: Chinese hamster ovary cells; CM: conditioned media; CTF: C-terminal fragment; DAPI: 4',6-diamidino-2-phenylindole; DMSO: dimethyl sulfoxide; DTP: Developmental Therapeutics Program; EGTA: ethylene glycol tetraacetic acid; ELISA: enzyme-linked immunosorbent assay; ER: endoplasmic reticulum; FA: formic acid; IgG: immunoglobulin G; LDH: lactate dehydrogenase; LTBP-1: latent transforming growth factor-binding protein-1; MS: multiple sclerosis; MTT: 3-(4, 5-dimethylthiazol-2-yl)-2, 5-diphenyltetrazolium bromide; NP40: nonidet-P40; PBS: phosphate-buffered saline; PFA: paraformaldehyde; PKA: protein kinase A; PVDF: polyvinylidene difluoride; RT: room temperature; TBI: traumatic brain injury; TBS-T: tris-buffered saline with Tween; TGFβ: transforming growth factor β.

## Competing interests

The authors declare that they have no competing interests.

## Authors' contributions

MKL is responsible for designing the overall study and writing the manuscript. CDH and DD equally contributed to the study by performing experiments. JPP and HW also performed experiments. KAP designed and performed the analytical part of the experiments. DM designed and performed the secretase experiments. AN designed the chemistry part of the experiments. All authors have read and approved the manuscript for publication.

## Pre-publication history

The pre-publication history for this paper can be accessed here:

http://www.biomedcentral.com/1741-7015/11/81/prepub
